# Confirmatory Composite Analysis

**DOI:** 10.3389/fpsyg.2018.02541

**Published:** 2018-12-13

**Authors:** Florian Schuberth, Jörg Henseler, Theo K. Dijkstra

**Affiliations:** ^1^Faculty of Engineering Technology, Chair of Product-Market Relations, University of Twente, Enschede, Netherlands; ^2^Nova Information Management School, Universidade Nova de Lisboa, Lisbon, Portugal; ^3^Faculty of Economics and Business, University of Groningen, Groningen, Netherlands

**Keywords:** artifacts, composite modeling, design research, Monte Carlo simulation study, structural equation modeling, theory testing

## Abstract

This article introduces confirmatory composite analysis (CCA) as a structural equation modeling technique that aims at testing composite models. It facilitates the operationalization and assessment of design concepts, so-called artifacts. CCA entails the same steps as confirmatory factor analysis: model specification, model identification, model estimation, and model assessment. Composite models are specified such that they consist of a set of interrelated composites, all of which emerge as linear combinations of observable variables. Researchers must ensure theoretical identification of their specified model. For the estimation of the model, several estimators are available; in particular Kettenring's extensions of canonical correlation analysis provide consistent estimates. Model assessment mainly relies on the Bollen-Stine bootstrap to assess the discrepancy between the empirical and the estimated model-implied indicator covariance matrix. A Monte Carlo simulation examines the efficacy of CCA, and demonstrates that CCA is able to detect various forms of model misspecification.

## 1. Introduction

Structural equation modeling with latent variables (SEM) comprises confirmatory factor analysis (CFA) and path analysis, thus combining methodological developments from different disciplines such as psychology, sociology, and economics, while covering a broad variety of traditional multivariate statistical procedures (Bollen, 1989; Muthén, 2002). It is capable of expressing theoretical concepts by means of multiple observable indicators to connect them via the structural model as well as to account for measurement error. Since SEM allows for statistical testing of the estimated parameters and even entire models, it is an outstanding tool for confirmatory purposes such as for assessing construct validity (Markus and Borsboom, 2013) or for establishing measurement invariance (Van de Schoot et al., 2012). Apart from the original maximum likelihood estimator, robust versions and a number of alternative estimators were also introduced to encounter violations of the original assumptions in empirical work, such as the asymptotic distribution free (Browne, 1984) or the two-stage least squares (2SLS) estimator (Bollen, 2001). Over time, the initial model has been continuously improved upon to account for more complex theories. Consequently, SEM is able to deal with categorical (Muthén, 1984) as well as longitudinal data (Little, 2013) and can be used to model non-linear relationships between the constructs (Klein and Moosbrugger, 2000).^1^

Researchers across many streams of science appreciate SEM's versatility as well as its ability to test common factor models. In particular, in the behavioral and social sciences, SEM enjoys wide popularity, e.g., in marketing (Bagozzi and Yi, 1988; Steenkamp and Baumgartner, 2000), psychology (MacCallum and Austin, 2000), communication science (Holbert and Stephenson, 2002), operations management (Shah and Goldstein, 2006), and information systems (Gefen et al., 2011),—to name a few. Additionally, beyond the realm of behavioral and social sciences, researchers have acknowledged the capabilities of SEM, such as in construction research (Xiong et al., 2015) or neurosciences (McIntosh and Gonzalez-Lima, 1994).

Over the last decades, the operationalization of the theoretical concept and the common factor has become more and more conflated such that hardly any distinction is made between the terms (Rigdon, 2012). Although the common factor model has demonstrated its usefulness for concepts of behavioral research such as traits and attitudes, the limitation of SEM to the factor model is unfortunate because many disciplines besides and even within social and behavioral sciences do not exclusively deal with behavioral concepts, but also with design concepts (so-called artifacts) and their interplay with behavioral concepts. For example Psychiatry: on the one hand it examines clinical relevant behavior to understand mental disorder, but on the other hand it also aims at developing mental disorder treatments (Kirmayer and Crafa, 2014). Table 1 displays further examples of disciplines investigating behavioral concepts and artifacts.

**Table 1:** Examples of behavioral concepts and artifacts across several disciplines.

**Discipline**	**Behavioral Concept**	**Design Concept (Artifact)**
Criminology	Criminal activity	Prevention strategy
	Lussier et al., 2005	Crowley, 2013
Ecology	Sediment contamination	Abiotic stress
	Malaeb et al., 2000	Grace et al., 2010
Education	Student's anxiety	Teacher development program
	Fong et al., 2016	Lee, 2005
Epidemiology	Nutritional Risk	Public health intervention
	Keller, 2006	Wight et al., 2015
Information Systems	Perceived ease of use	User-interface design
	Venkatesh et al., 2003	Vance et al., 2015
Marketing	Brand attitude	Marketing mix
	Spears and Singh, 2004	Borden, 1964

Typically, the common factor model is used to operationalize behavioral concepts, because it is well matched with the general understanding of measurement (Sobel, 1997). It assumes that each observable indicator is a manifestation of the underlying concept that is regarded as their *common cause* (Reichenbach, 1956), and therefore fully explains the covariation among its indicators. However, for artifacts the idea of measurement is unrewarding as they are rather constructed to fulfill a certain purpose. To account for the constructivist character of the artifact, the composite has been recently suggested for its operationalization in SEM (Henseler, 2017). A composite is a weighted linear combination of observable indicators, and therefore in contrast to the common factor model, the indicators do not necessarily share a common cause.

At present, the validity of composite models cannot be systematically assessed. Current approaches are limited to assessing the indicators' collinearity (Diamantopoulos and Winklhofer, 2001) and their relations to other variables in the model (Bagozzi, 1994). A rigorous test of composite models in analogy to CFA does not exist so far. Not only does this situation limit the progress of composite models, it also represents an unnecessary weakness of SEM as its application is mainly limited to behavioral concepts. For this reason, we introduce confirmatory composite analysis (CCA) wherein the concept, i.e., the artifact, under investigation is modeled as a composite. In this way, we make SEM become accessible to a broader audience. We show that the composite model relaxes some of the restrictions imposed by the common factor model. However, it still provides testable constraints, which makes CCA a full-fledged method for confirmatory purposes. In general, it involves the same steps as CFA or SEM, without assuming that the underlying concept is necessarily modeled as a common factor.

While there is no exact instruction on how to apply SEM, a general consensus exists that SEM and CFA comprise at least the following four steps: model specification, model identification, model estimation, and model assessment (e.g., Schumacker and Lomax, 2009, Chap. 4). To be in line with this proceeding, the remainder of the paper is structured as follows: Section 2 introduces the composite model providing the theoretical foundation for the CCA and how the same can be specified; Section 3 considers the issue of identification in CCA and states the assumptions as being necessary to guarantee the unique solvability of the composite model; Section 4 presents one approach that can be used to estimate the model parameters in the framework of CCA; Section 5 provides a test for the overall model fit to assess how well the estimated model fits the observed data; Section 6 assesses the performance of this test in terms of a Monte Carlo simulation and presents the results; and finally, the last section discusses the results and gives an outlook for future research. A brief example on how to estimate and assess a composite model within the statistical programming environment R is provided in the Supplementary Material.

## 2. Specifying Composite Models

Composites have a long tradition in multivariate data analysis (Pearson, 1901). Originally, they are the outcome of dimension reduction techniques, i.e., the mapping of the data to a lower dimensional space. In this respect, they are designed to capture the most important characteristics of the data as efficiently as possible. Apart from dimension reduction, composites can serve as proxies for concepts (MacCallum and Browne, 1993). In marketing research, Fornell and Bookstein (1982) recognized that certain concepts like marketing mix or population change are not appropriately modeled by common factors and instead employed a composite to operationalize these concepts. In the recent past, more and more researchers recognized composites as a legitimate approach to operationalize concepts, e.g., in marketing science (Diamantopoulos and Winklhofer, 2001; Rossiter, 2002), business research (Diamantopoulos, 2008), environmental science (Grace and Bollen, 2008), and in design research (Henseler, 2017).

In social and behavioral sciences, concepts are often understood as ontological entities such as abilities or attitudes, which rests on the assumption that the concept of interest exists in nature, regardless of whether it is the subject of scientific examination. Researchers follow a positivist research paradigm assuming that existing concepts can be measured.

In contrast, design concepts can be conceived as artifacts, i.e., objects designed to serve explicit goal(s) (Simon, 1969). Hence, they are inextricably linked to purposefulness, i.e., teleology (Horvath, 2004; Baskerville and Pries-Heje, 2010; Møller et al., 2012). This way of thinking has its origin in constructivist epistemology. The epistemological distinction between the ontological and constructivist nature of concepts has important implications when modeling the causal relationships among the concepts and their relationships to the observable indicators.

**Figure 1:**
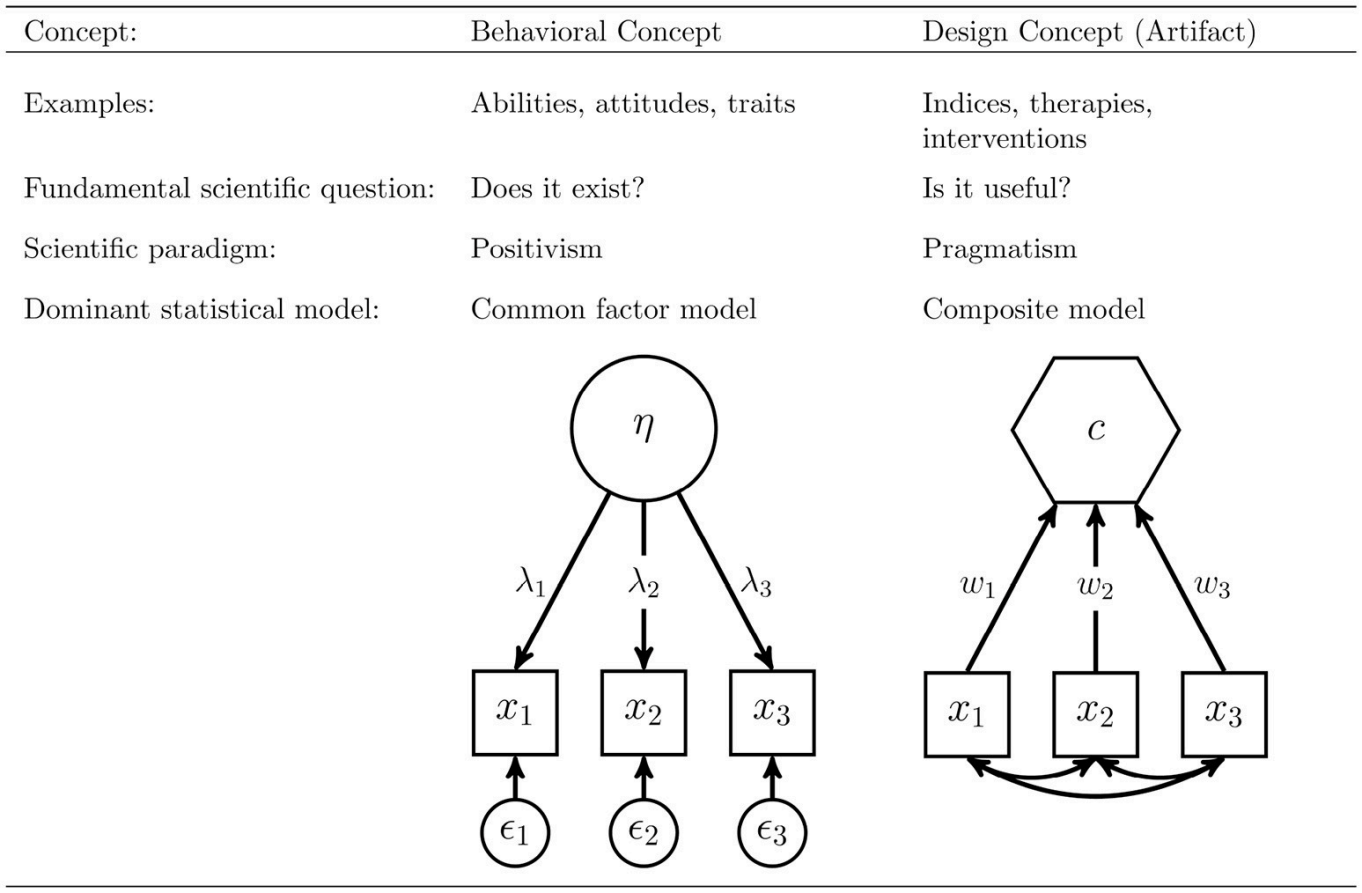
Two types of concepts: behavioral concepts vs. artifacts.

To operationalize behavioral concepts, the common factor model is typically used. It seeks to explore whether a certain concept exists by testing if collected measures of a concept are consistent with the assumed nature of that concept. It is based on the principle of common cause (Reichenbach, 1956), and therefore assumes that all covariation within a block of indicators can be fully explained by the underlying concept. On the contrary, the composite model can be used to model artifacts as a linear combination of observable indicators. In doing so, it is more pragmatic in the sense that it examines whether a built artifact is useful at all. Figure 1 summarizes the differences between behavioral concepts and artifacts and their operationalization in SEM.

In the following part, we present the theoretical foundation of the composite model. Although the formal development of the composite model and the *composite factor model* (Henseler et al., 2014), were already laid out by Dijkstra (2013, 2015), it has not been put into a holistic framework yet. In the following, it is assumed that each artifact is modeled as a composite *c*_*j*_ with *j* = 1, …, *J*.^2^ By definition, a composite is completely determined by a unique block of *K*_*j*_ indicators, ${\text{x}}_{j}^{\prime }=\left(\begin{array}{ccc} x_{j1} & \ldots & x_{jK_{j}} \end{array}\right)$, $c_{j}={\text{w}}_{j}^{\prime }{\text{x}}_{j}$.

The weights of block *j* are included in the column vector ***w***_*j*_ of length *K*_*j*_. Usually, each weight vector is scaled to ensure that the composites have unit variance (see also Section 3). Here, we assume that each indicator is connected to only one composite. The theoretical covariance matrix **Σ** of the indicators can be expressed as a partitioned matrix as follows:

(1) $$\begin{array}{l} \Sigma =\left(\begin{array}{llll} \Sigma_{11} & \Sigma_{12} & \ldots & \Sigma_{1J}\\ \; & \Sigma_{22} & \ldots & \Sigma_{2J}\\ \; & \; & \ddots & \vdots \\ \; & \; & \; & \Sigma_{JJ} \end{array}\right) \end{array}$$

The intra-block covariance matrix **Σ**_*jj*_ of dimension *K*_*j*_ × *K*_*j*_ is unconstrained and captures the covariation between the indicators of block *j*; thus, this effectively allows the indicators of one block to freely covary. Moreover, it can be shown that the indicator covariance matrix is positive-definite if and only if the following two conditions hold: (i) all intra-block covariance matrices are positive-definite, and (ii) the covariance matrix of the composite is positive-definite (Dijkstra, 2015, 2017). The covariances between the indicators of block *j* and *l* are captured in the inter-block covariance matrix **Σ**_*jl*_, with *j* ≠ *l* of dimension *K*_*j*_ × *K*_*l*_. However, in contrast to the intra-block covariance matrix, the inter-block covariance matrix is constrained, since by assumption, the composites carry all information between the blocks:

(2) $$\begin{array}{l} \Sigma_{jl}=\rho_{jl}\Sigma_{jj}{\text{w}}_{j}{\text{w}}_{l}^{\prime }\Sigma_{ll}=\rho_{jl}\lambda_{j}\lambda_{l}^{\prime }, \end{array}$$

where $\rho_{jl}={\text{w}}_{j}^{\prime }\Sigma_{jl}{\text{w}}_{l}$ equals the correlation between the composites *c*_*j*_ and *c*_*l*_. The vector **λ**_*j*_ = **Σ**_*jj*_***w***_*j*_ of length *K*_*j*_ contains the composite loadings, which are defined as the covariances between the composite *c*_*j*_ and the associated indicators ***x***_*j*_. Equation 2 is highly reminiscent of the corresponding equation where all concepts are modeled as common factors instead of composites. In a common factor model, the vector **λ**_*j*_ captures the covariances between the indicators and its connected common factor, and ρ_*jl*_ represents the correlation between common factor *j* and *l*. Hence, both models show the rank-one structure for the covariance matrices between two indicator blocks.

**Figure 2:**
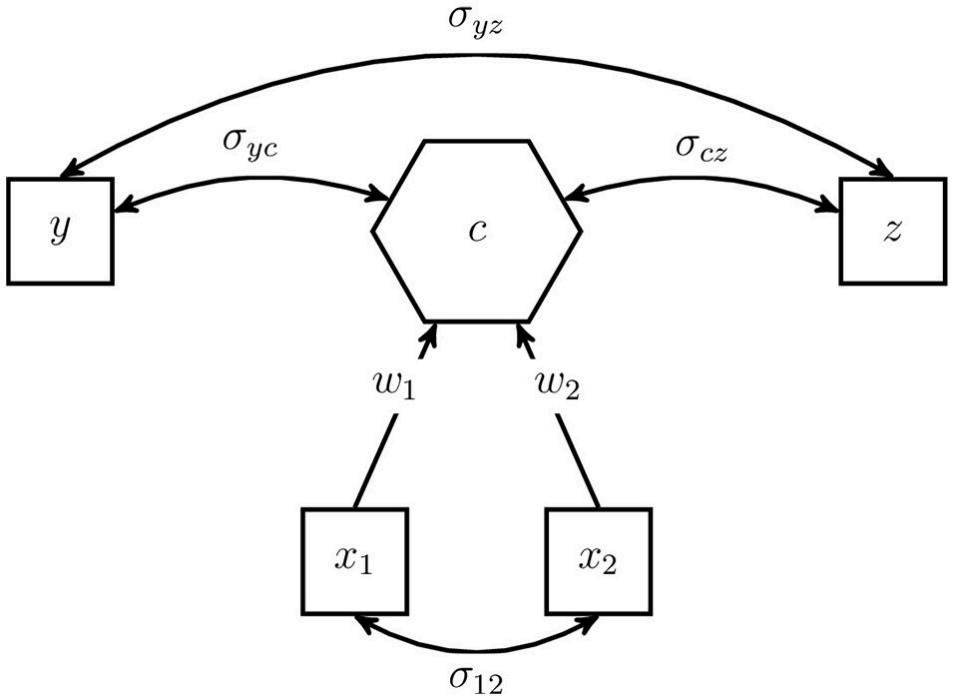
Example of a composite model.

Although the intra-block covariance matrices of the indicators **Σ**_*jj*_ are not restricted, we emphasize that the composite model is still a model from the point of view of SEM. It assumes that all information between the indicators of two different blocks is conveyed by the composite(s), and therefore, it imposes rank-one restrictions on the inter-block covariance matrices of the indicators (see Equation 2). These restrictions can be exploited for testing the overall model fit (see Section 5). It is emphasized that the weights ***w***_*j*_ producing these matrices are the same across all inter-block covariance matrices **Σ**_*jl*_ with *l* = 1, …, *J* and *l* ≠ *j*. Figure 2 illustrates an example of a composite model.

The artifact under investigation is modeled as the composite *c*, illustrated by a hexagon, and the observable indicators are represented by squares. The unconstrained covariance σ_12_ between the indicators of block $x\prime =(\begin{array}{cc} x_{1} & x_{2} \end{array})$ forming the composite is highlighted by a double-headed arrow.

The observable variables *y* and *z* do not form the composite. They are allowed to freely covary among each other as well as with the composite. For example, they can be regarded as antecedents or consequences of the modeled artifact.

**Figure 3:**
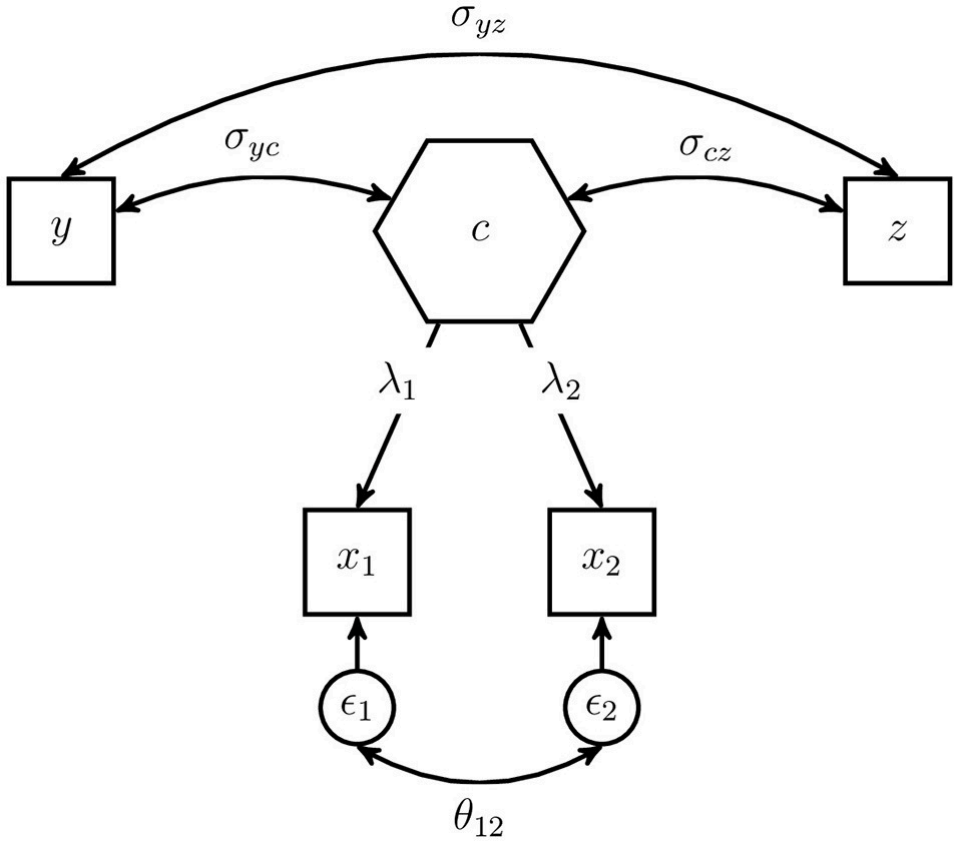
Example of a composite model displayed as composite factor model.

To emphasize the difference between the composite model and the common factor model typically used in CFA, we depict the composite model as *composite factor* model (Dijkstra, 2013; Henseler et al., 2014). The composite factor model has the same model-implied indicator covariance matrix as the composite model, but the deduction of the model-implied covariances and the comparison to the common factor is more straightforward. Figure 3 shows the same model as Figure 2 but in terms of a *composite factor* representation.

The composite loading λ_*i*_, *i* = 1, 2 captures the covariance between the indicator *x*_*i*_ and the composite *c*. In general, the error terms are included in the vector **ϵ**, explaining the variance of the indicators and the covariances between the indicators of one block, which are not explained by the composite factor. As the composite model does not restrict the covariances between the indicators of one block, the error terms are allowed to freely covary. The covariations among the error terms as well as their variances are captured in matrix **Θ**. The model-implied covariance matrix of the example composite model can be displayed as follows:

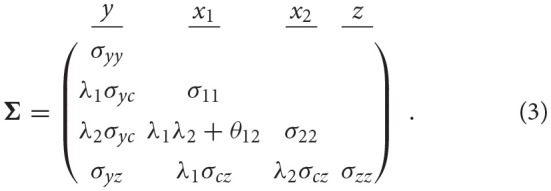



In comparison to the same model using a common factor instead of a composite, the composite model is less restrictive as it allows all error terms of one block to be correlated, which leads to a more general model (Henseler et al., 2014). In fact, the common factor model is always nested in the composite model since it uses the same restriction as the composite model; but additionally, it assumes that (some) covariances between the error terms of one block are restricted (usually to zero). Under certain conditions, it is possible to rescale the intra- and inter-block covariances of a composite model to match those of a common factor model (Dijkstra, 2013; Dijkstra and Henseler, 2015).

## 3. Identifying Composite Models

Like in SEM and CFA, model identification is an important issue in CCA. Since analysts can freely specify their models, it needs to be ensured that the model parameters have a unique solution (Bollen, 1989, Chap. 8). Therefore, model identification is necessary to obtain consistent parameter estimates and to reliably interpret them (Marcoulides and Chin, 2013).

In general, the following three states of model identification can be distinguished: under-identified, just-identified, and over-identified.^3^ An under-identified model, also known as not-identified model, offers several sets of parameters that are consistent with the model constraints, and thus, no unique solution for the model parameters exists. Therefore, only questionable conclusions can be drawn. In contrast, a just-identified model provides a unique solution for the model parameters and has the same number of free parameters as non-redundant elements of the indicator covariance matrix (degrees of freedom (df) are 0). In empirical analysis, such models cannot be used to evaluate the overall model fit since they perfectly fit the data. An over-identified model also has a unique solution; however, it provides more non-redundant elements of the indicator covariance matrix than model parameters (df > 0). This can be exploited in empirical studies for assessing the overall model fit, as these constraints should hold for a sample within the limits of sampling error if the model is valid.

A necessary condition for ensuring identification is to normalize each weight vector. In doing so, we assume that all composites are scaled to have a unit variance, ${\text{w}}_{j}^{\prime }\Sigma_{jj}{\text{w}}_{j}=1$.^4^ Besides the scaling of the composite, each composite must be connected to at least one composite or one variable not forming a composite. As a result, at least one inter-block covariance matrix **Σ**_*jl*_, *l* = 1, …, *J* with *l* ≠ *j* satisfies the rank-one condition. Along with the normalization of the weight vectors, all model parameters can be uniquely retrieved from the indicator covariance matrix since there is a non-zero inter-block covariance matrix for every loading vector. Otherwise, if a composite *c*_*i*_ is isolated in the nomological network, all inter-block covariances **Σ**_*jl*_, *l* = 1, …, *J* with *l* ≠ *j*, belonging to this composite are of rank zero, and thus, the weights forming this composite cannot be uniquely retrieved. Although the non-isolation condition is required for identification, it also matches the idea of an artifact that is designed to fulfill a certain purpose. Without considering the artifact's antecedents and/or consequences, the artifact's purposefulness cannot be judged.

In the following part, we give a description on how the number of degrees of freedom is counted in case of the composite model.^5^ It is given by the difference between the number of non-redundant elements of the indicator population covariance matrix **Σ** and the number of free parameters in the model. The number of free model parameters is given by the number of covariances among the composites, the number of covariances between composites and indicators not forming a composite, the number of covariances among indicators not forming a composite, the number of non-redundant off-diagonal elements of each intra-block covariance matrix, and the number of weights. Since we fix composite variances to one, one weight of each block can be expressed by the remaining ones of this block. Hence, we regain as many degrees of freedom as fixed composite variances, i.e., as blocks in the model. Equation 4 summarizes the way of determining the number of degrees of freedom of a composite model.

(4) $$\begin{array}{l} \text{df}=\;\text{number of non-redundant off-diagonal elements of the}\\ \text{ indicator covariance matrix}\\ \;-\;\text{number of free correlations among the composites}\\ \;-\;\text{number of free covariances between the composites and}\\ \text{ indicators not forming a composite}\\ \;-\;\text{number of covariances among the indicators not forming}\\ \text{ a composite}\\ \;-\;\text{number of free non-redundant off-diagonal elements of}\\ \text{ each intra-block covariance matrix}\\ \;-\;\text{number of weights}\\ \;+\;\text{number of blocks} \end{array}$$

To illustrate our approach to calculating the number of degrees of freedom, we consider the composite model presented in Figure 2. As described above, the model consists of four (standardized) observable variables; thus, the indicator correlation matrix has six non-redundant off-diagonal elements. The number of free model parameters is counted as follows: no correlations among the composites as the models consists of only one composite, two correlations between the composite and the observable variables not forming a composite (σ_*yc*_ and σ_*cz*_), one correlation between the variables not forming a composite (σ_*yz*_), one non-redundant off-diagonal of the intra-block correlation matrix (σ_12_), and two weights (*w*_1_ and *w*_2_) minus one, the number of blocks. As a result, we obtain the number of degrees of freedom as follows: *df* = 6−0−2−1−1−2 + 1 = 1. Once identification of the composite model is ensured, in a next step the model can be estimated.

## 4. Estimating Composite Models

The existing literature provides various ways of constructing composites from blocks of indicators. The most common among them are principal component analysis (PCA, Pearson, 1901), linear discriminant analysis (LDA, Fisher, 1936), and (generalized) canonical correlation analysis ((G)CCA, Hotelling, 1936; Kettenring, 1971). All these approaches seek composites that “best” explain the data and can be regarded as *prescriptions* for dimension reduction (Dijkstra and Henseler, 2011). Further approaches are partial least squares path modeling (PLS-PM, Wold, 1975), regularized general canonical correlation analysis (RGCCA, Tenenhaus and Tenenhaus, 2011), and generalized structural component analysis (GSCA, Hwang and Takane, 2004). The use of predefined weights is also possible.

We follow Dijkstra (2010) and apply GCCA in a first step to estimate the correlation between the composites.^6^ In the following part, we give a brief description of GCCA. The vector of indicators ***x*** of length *K* is split up into *J* subvectors ***x***_*j*_, so called blocks, each of dimension (*K*_*j*_ × 1) with *j* = 1, …, *J*. We assume that the indicators are standardized to have means of zero and unit variances. Moreover, each indicator is connected to one composite only. Hence, the correlation matrix of the indicators can be calculated as **Σ** = E(***xx***′) and the intra-block correlation matrix as $\Sigma_{jj}=\text{E}\left({\text{x}}_{j}{\text{x}}_{j}^{\prime }\right)$. Moreover, the correlation matrix of the composites $c_{j}={\text{x}}_{j}^{\prime }{\text{w}}_{j}$ is calculated as follows: $\Sigma_{\text{c}}=\text{E}\left(\text{c}{\text{c}}^{\prime }\right)$. In general, GCCA chooses the weights to maximize the correlation between the composites. In doing so, GCCA offers the following options: *sumcor, maxvar, ssqcor, minvar*, and *genvar*.^7^

In the following part, we use *maxvar* under the constraint that each composite has a unit variance, ${\text{w}}_{j}^{\prime }\Sigma_{jj}{\text{w}}_{j}=1$, to estimate the weights, the composites, and the resulting composite correlations.^8^ In doing so, the weights are chosen to maximize the largest eigenvalue of the composite correlation matrix. Thus, the total variation of the composites is explained as well as possible by one underlying “principal component,” and the weights to form the composite *c*_*j*_ are calculated as follows (Kettenring, 1971):

(5) $$\begin{array}{l} {\text{w}}_{j}=\Sigma_{jj}^{-\frac{1}{2}}{\tilde{\text{a}}}_{j}/\sqrt{{\tilde{\text{a}}}_{j}^{\prime }{\tilde{\text{a}}}_{j}}. \end{array}$$

The subvector ${\tilde{\text{a}}}_{j}$, of length *J*, corresponds to the largest eigenvalue of the matrix $\Sigma_{D}^{-\frac{1}{2}}\Sigma \Sigma_{D}^{-\frac{1}{2}}$, where the matrix **Σ**_*D*_, of dimension *J* × *J*, is a block-diagonal matrix containing the intra-block correlation matrices **Σ**_*jj*_, *j* = 1, …, *J* on its diagonal. To obtain the estimates of the weights, the composites, and their correlations, the population matrix **Σ** is replaced by its empirical counterpart ***S***.

## 5. Assessing Composite Models

### 5.1. Tests of Overall Model Fit

In CFA and factor-based SEM, a test for overall model fit has been naturally supplied by the maximum-likelihood estimation in the form of the chi-square test (Jöreskog, 1967), while maxvar lacks in terms of such a test. In the light of this, we propose a combination of a bootstrap procedure with several distance measures to statistically test how well the assumed composite model fits to the collected data.

The existing literature provides several measures with which to assess the discrepancy between the perfect fit and the model fit. In fact, every distance measure known from CFA can be used to assess the overall fit of a composite model. They all capture the discrepancy between the sample covariance matrix ***S*** and the estimated model-implied covariance matrix $\hat{\Sigma }=\Sigma \left(\hat{\theta }\right)$ of the indicators. In our study, we consider the following three distance measures: squared Euclidean distance (*d*_*L*_), geodesic distance (*d*_*G*_), and standardized root mean square residual (SRMR).

The squared Euclidean distance between the sample and the estimated model-implied covariance matrix is calculated as follows:

(6) $$\begin{array}{l} d_{\text{L}}=\frac{1}{2}\overset{K}{\underset{i=1}{\sum }}\overset{K}{\underset{j=1}{\sum }}{\left(s_{ij}-{\hat{\sigma }}_{ij}\right)}^{2}, \end{array}$$

where *K* is the total number of indicators, and *s*_*ij*_ and ${\hat{\sigma }}_{ij}$ are the elements of the sample and the estimated model-implied covariance matrix, respectively. It is obvious that the squared Euclidean distance is zero for a perfectly fitting model, $\hat{\Sigma }=\text{S}$.

Moreover, the geodesic distance stemming from a class of distance functions proposed by Swain (1975) can be used to measure the discrepancy between the sample and estimated model-implied covariance matrix. It is given by the following:

(7) $$\begin{array}{l} d_{\text{G}}=\frac{1}{2}\overset{K}{\underset{i=1}{\sum }}{\left(\text{log}\left(\varphi_{i}\right)\right)}^{2}, \end{array}$$

where φ_*i*_ is the *i*-th eigenvalue of the matrix ${\text{S}}^{-1}\hat{\Sigma }$ and *K* is the number of indicators. The geodesic distance is zero when and only when all eigenvalues equal one, i.e., when and only when the fit is perfect.

Finally, the SRMR (Hu and Bentler, 1999) can be used to assess the overall model fit. The SRMR is calculated as follows:

(8) $$\begin{array}{l} \text{SRMR}=\sqrt{\left[2\overset{K}{\underset{i=1}{\sum }}\overset{i}{\underset{j=1}{\sum }}{\left(\left(s_{ij}-{\hat{\sigma }}_{ij}\right)/\left(s_{ii}s_{jj}\right)\right)}^{2}\right]/\left(K\left(K+1\right)\right)}, \end{array}$$

where *K* is the number of indicators. It reflects the average discrepancy between the empirical and the estimated model-implied correlation matrix. Thus, for a perfectly fitting model, the SRMR is zero, as ${\hat{\sigma }}_{ij}$ equals *s*_*ij*_.

Since all distance measures considered are functions of the sample covariance matrix, a procedure proposed by Beran and Srivastava (1985) can be used to test the overall model fit: *H*_0_ : **Σ** = **Σ**(**θ**).^9^ The reference distribution of the distance measures as well as the critical values are obtained from the transformed sample data as follows:

(9) $$\begin{array}{l} \text{X}{\text{S}}^{-\frac{1}{2}}{\hat{\Sigma }}^{\frac{1}{2}}, \end{array}$$

where the data matrix ***x*** of dimension (*N* × *K*) contains the *N* observations of all *K* indicators. This transformation ensures that the new dataset satisfies the null hypothesis; i.e., the sample covariance matrix of the transformed dataset equals the estimated model-implied covariance matrix. The reference distribution of the distance measures is obtained by bootstrapping from the transformed dataset. In doing so, the estimated distance based on the original dataset can be compared to the critical value from the reference distribution (typically the empirical 95% or 99% quantile) to decide whether the null hypothesis, *H*_0_ : **Σ** = **Σ**(**θ**) is rejected (Bollen and Stine, 1992).

### 5.2. Fit Indices for Composite Models

In addition to the test of overall model fit, we provide some fit indices as measures of the overall model fit. In general, fit indices can indicate whether a model is misspecified by providing an absolute value of the misfit; however, we advise using them with caution as they are based on heuristic rules-of-thumb rather than statistical theory. Moreover, it is recommended to calculate the fit indices based on the indicator correlation matrix instead of the covariance matrix.

The standardized root mean square residual (SRMR) was already introduced as a measure of overall model fit (Henseler et al., 2014). As described above, it represents the average discrepancy between the sample and the model-implied indicator correlation matrix. Values below 0.10 and, following a more conservative view, below 0.08 indicate a good model fit (Hu and Bentler, 1998). However, these threshold values were proposed for common factor models and their usefulness for composite models needs to be investigated.

Furthermore, the normed fit index (NFI) is suggested as a measure of goodness of fit (Bentler and Bonett, 1980). It measures the relative discrepancy between the fit of the baseline model and the fit of the estimated model. In this context, a model where all indicators are assumed to be uncorrelated (the model-implied correlation matrix equals the unit matrix) can serve as a baseline model (Lohmöller, 1989, Chap. 2.4.4). To assess the fit of the baseline model and the estimated model, several measures can be used, e.g., the log likelihood function used in CFA or the geodesic distance. Values of the NFI close to one imply a good model fit. However, cut-off values still need to be determined.

Finally, we suggest considering the root mean square residual covariance of the outer residuals (RMS_theta_) as a further fit index (Lohmöller, 1989). It is defined as the square root of the average residual correlations. Since the indicators of one block are allowed to be freely correlated, the residual correlations within a block should be excluded and only the residual correlations across the blocks should be taken into account during its calculation. Small values close to zero for the RMS_theta_ indicate a good model fit. However, threshold values still need to be determined.

## 6. A Monte Carlo Simulation

**Figure 4:**
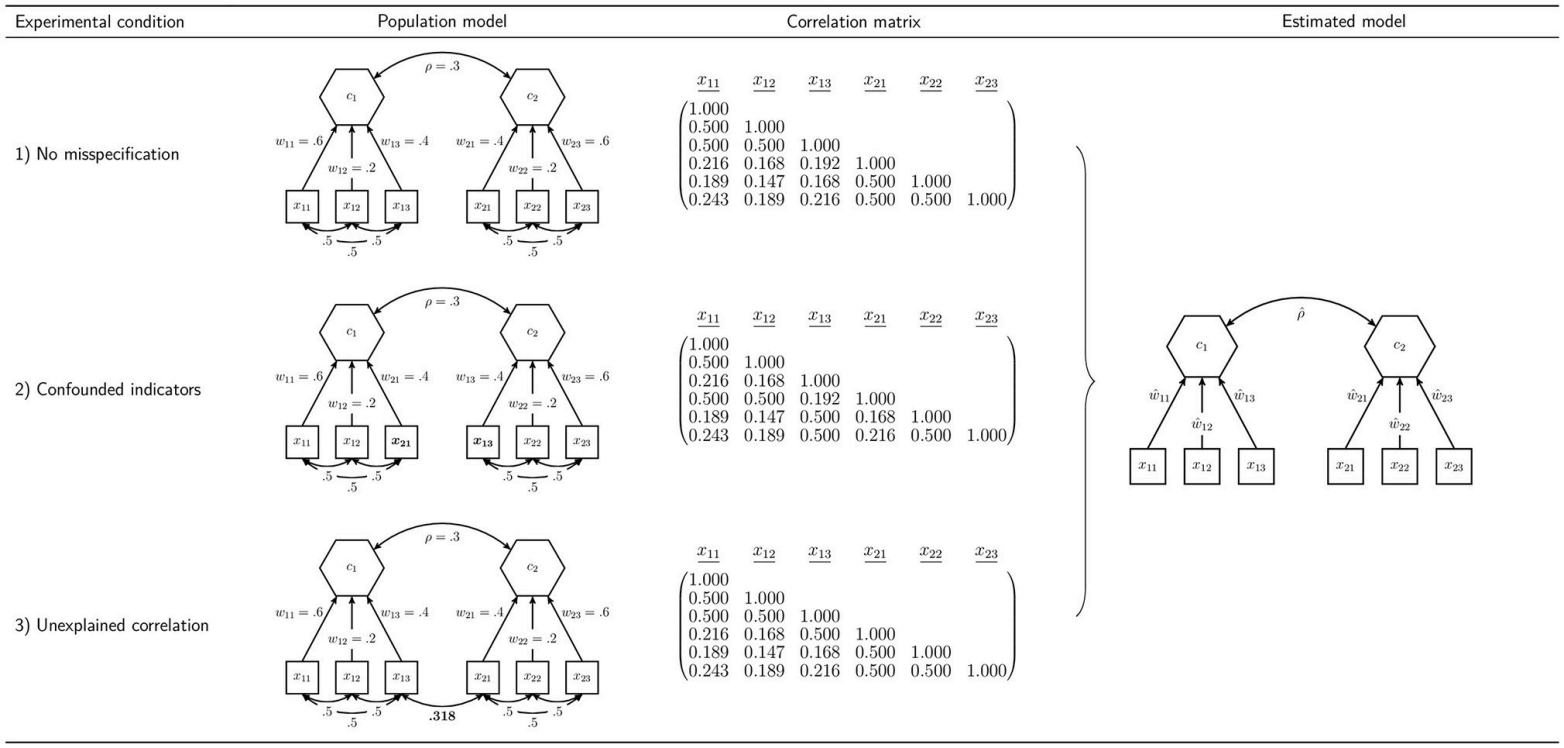
Simulation design for the model containing two composites.

**Figure 5:**
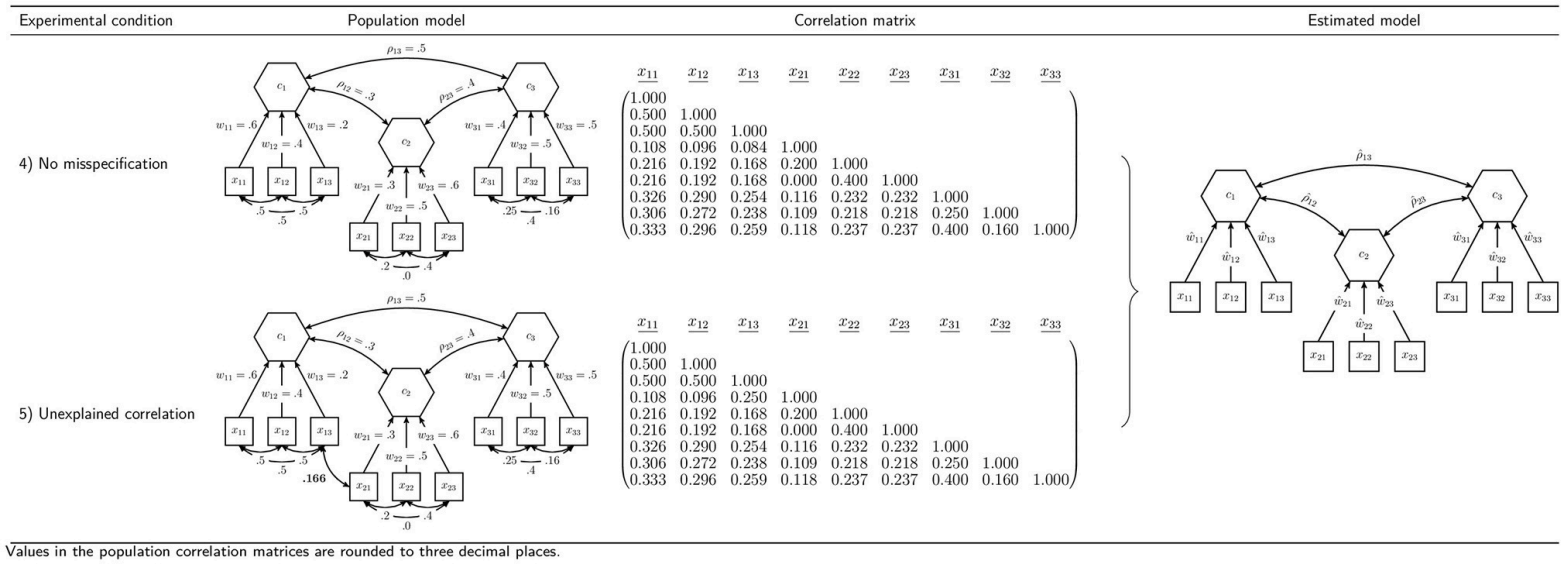
Simulation design for the model containing three composites.

In order to assess our proposed procedure of statistically testing the overall model fit of composite models and to examine the behavior of the earlier presented discrepancy measures, we conduct a Monte Carlo simulation. In particular, we investigate the type I error rate (false positive rate) and the power, which are the most important characteristics of a statistical test. In designing the simulation, we choose a number of concepts used several times in the literature to examine the performance of fit indices and tests of overall model fit in CFA: a model containing two composites and a model containing three composites (Hu and Bentler, 1999; Heene et al., 2012). To investigate the power of the test procedure, we consider various misspecifications of these models. Figures 4 and 5 summarize the conditions investigated in our simulation study.

### 6.1. Model Containing Two Composites

All models containing two composites are estimated using the specification illustrated in the last column of Figure 4. The indicators *x*_11_ to *x*_13_ are specified to build composite *c*_1_, while the remaining three indicators build composite *c*_2_. Moreover, the composites are allowed to freely correlate. The parameters of interest are the correlation between the two composites, and the weights, *w*_11_ to *w*_23_. As column “Population model” of Figure 4 shows, we consider three types of population models with two composites.

#### 6.1.1. Condition 1: No Misspecification

First, in order to examine whether the rejection rates of the test procedure are close to the predefined significance level in cases in which the null hypothesis is true, a population model is considered that has the same structure as the specified model. The correlation between the two composites is set to ρ = 0.3 and the composites are formed by its connected standardized indicators as follows: $c_{i}={\text{x}}_{i}^{\prime }{\text{w}}_{i}$ with *i* = 1, 2, where ${\text{w}}_{1}^{\prime }=\left(\begin{array}{ccc} 0.6 & 0.2 & 0.4 \end{array}\right)$ and ${\text{w}}_{2}^{\prime }=\left(\begin{array}{ccc} 0.4 & 0.2 & 0.6 \end{array}\right)$. All correlations between the indicators of one block are set to 0.5, which leads to the population correlation matrix given in Figure 4.

#### 6.1.2. Condition 2: Confounded Indicators

The second condition is used to investigate whether the test procedure is capable of detecting misspecified models. It presents a situation where the researcher falsely assigns two indicators to wrong constructs. The correlation between the two composites and the weights are the same as in population model 1: ρ = 0.3, ${\text{w}}_{1}^{\prime }=\left(\begin{array}{ccc} 0.6 & 0.2 & 0.4 \end{array}\right)$, and ${\text{w}}_{2}^{\prime }=\left(\begin{array}{ccc} 0.4 & 0.2 & 0.6 \end{array}\right)$. However, in contrast to population model 1, the indicators *x*_13_ and *x*_21_ are interchanged. Moreover, the correlations among all indicators of one block are 0.5. The population correlation matrix of the second model is presented in Figure 4.

#### 6.1.3. Condition 3: Unexplained Correlation

The third condition is chosen to further investigate the capabilities of the test procedure to detect misspecified models. It shows a situation where the correlation between the two indicators *x*_13_ and *x*_21_ is not fully explained by the two composites.^10^ As in the two previously presented population models, the two composites have a correlation of ρ = 0.3. The correlations among the indicators of one block are set to 0.5, and the weights for the construction of the composites are set to ${\text{w}}_{1}^{\prime }=\left(\begin{array}{ccc} 0.6 & 0.2 & 0.4 \end{array}\right)$, and ${\text{w}}_{2}^{\prime }=\left(\begin{array}{ccc} 0.4 & 0.2 & 0.6 \end{array}\right)$. The population correlation matrix of the indicators is presented in Figure 4.

### 6.2. Model Containing Three Composites

Furthermore, we investigate a more complex model consisting of three composites. Again, each composite is formed by three indicators, and the composites are allowed to freely covary. The column “Estimated model” of Figure 5 illustrates the specification to be estimated in case of three composites. We assume that the composites are built as follows: $c_{1}={\text{x}}_{1}^{\prime }{\text{w}}_{1}$, $c_{2}={\text{x}}_{2}^{\prime }{\text{w}}_{2}$, and $c_{3}={\text{x}}_{3}^{\prime }{\text{w}}_{3}$. Again, we examine two different population models.

#### 6.2.1. Condition 4: No Misspecification

The fourth condition is used to further investigate whether the rejection rates of the test procedure are close to the predefined significance level in cases in which the null hypothesis is true. Hence, the structure of the fourth population model matches the specified model. All composites are assumed to be freely correlated. In the population, the composite correlations are set to ρ_12_ = 0.3, ρ_13_ = 0.5, and ρ_23_ = 0.4. Each composite is built by three indicators using the following population weights: ${\text{w}}_{1}^{\prime }=\left(\begin{array}{ccc} 0.6 & 0.4 & 0.2 \end{array}\right)$, ${\text{w}}_{2}^{\prime }=\left(\begin{array}{ccc} 0.3 & 0.5 & 0.6 \end{array}\right)$, and ${\text{w}}_{3}^{\prime }=\left(\begin{array}{ccc} 0.4 & 0.5 & 0.5 \end{array}\right)$. The indicator correlations of each block can be read from Figure 5. The indicator correlation matrix of population model 4 is given in Figure 5.

#### 6.2.2. Condition 5: Unexplained Correlation

In the fifth condition, we investigate a situation where the correlation between two indicators is not fully explained by the underlying composites, similar to what is observed in Condition 3. Consequently, population model 5 does not match the model to be estimated and is used to investigate the power of the overall model test. It equals population model 4 with the exception that the correlation between the indicators *x*_13_ and *x*_21_ is only partly explained by the composites. Since the original correlation between these indicators is 0.084, a correlation of 0.25 presents only a weak violation. The remaining model stays untouched. The population correlation matrix is illustrated in Figure 5.

### 6.3. Further Simulation Conditions and Expectations

To assess the quality of the proposed test of the overall model fit, we generate 10,000 standardized samples from the multivariate normal distribution having zero means and a covariance matrix according to the respective population model. Moreover, we vary the sample size from 50 to 1,450 observations (with increments of 100) and the significance level α from 1% to 10%. To obtain the reference distribution of the discrepancy measures considered, 200 bootstrap samples are drawn from the transformed and standardized dataset. Each dataset is used in the maxvar procedure to estimate the model parameters.

All simulations are conducted in the statistical programming environment R (R Core Team, 2016). The samples are drawn from the multivariate normal distribution using the *mvrnorm* function of the *MASS* packages (Venables and Ripley, 2002). The results for the test of overall model fit are obtained by user-written functions^11^ and the *matrixpls* package (Rönkkö, 2016).

Since population models 1 and 4 fit the respective specification, we expect rejection rates close to the predefined levels of significance α. Additionally, we expect that for an increasing sample size, the predefined significance level is kept with more precision. For population model 2, 3, and 5, much larger rejection rates are expected as these population models do not match the respective specification. Moreover, we expect that the power of the test to detect misspecifications would increase along with a larger sample size. Regarding the different discrepancy measures, we have no expectations, only that the squared Euclidean distance and the SRMR should lead to identical results. For standardized datasets, the only difference is a constant factor that does not affect the order of the observations in the reference distribution and, therefore, does not affect the decision about the null hypothesis.

### 6.4. Results

**Figure 6:**
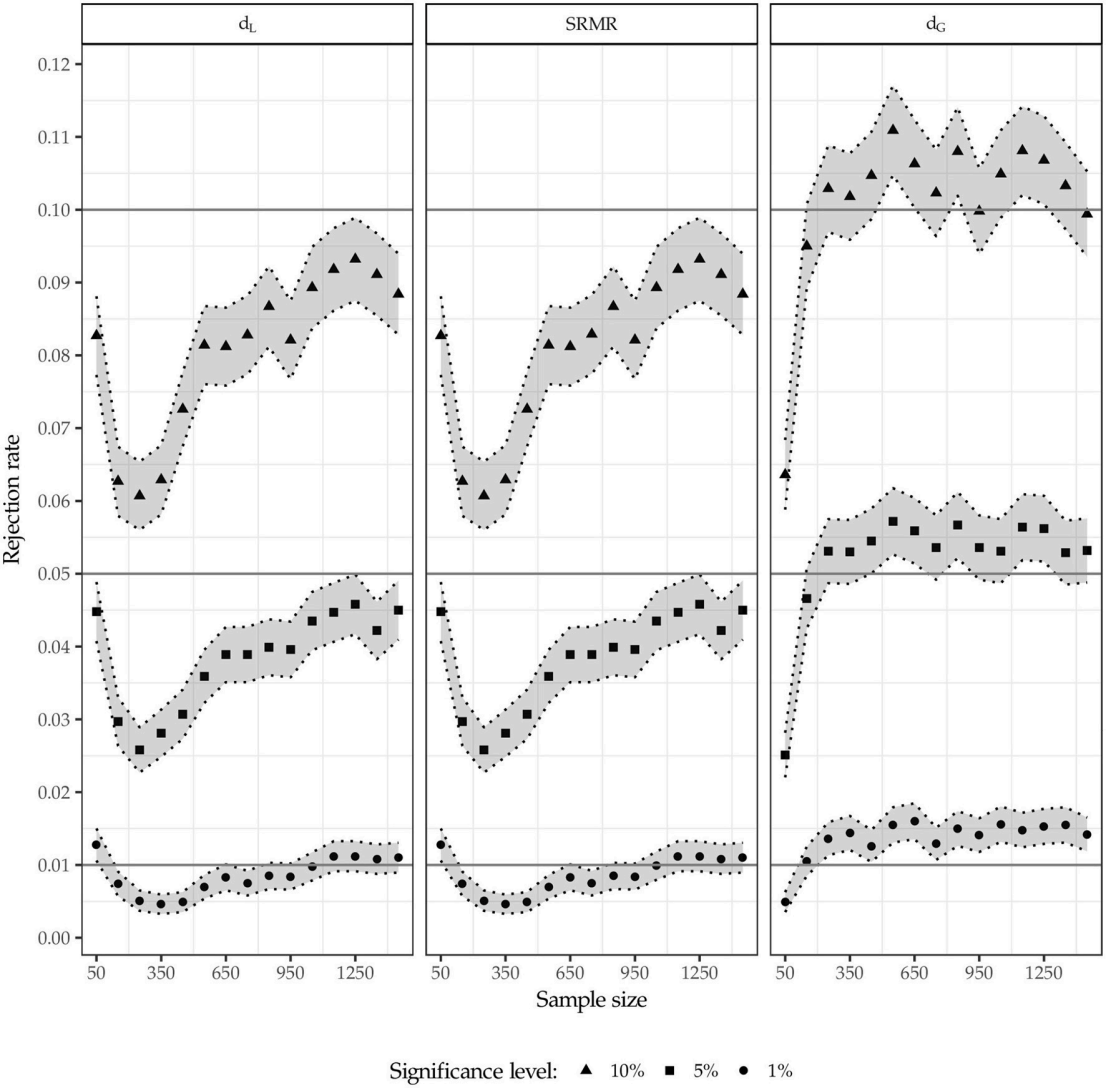
Rejection rates for population model 1.

Figure 6 illustrates the rejection rates for population model 1 i.e., no misspecification. Besides the rejection rates, the figure also depicts the 95% confidence intervals (shaded area) constructed around the rejection rates to clarify whether a rejection rate is significantly different from the predefined significance level.^12^

First, as expected, the squared Euclidean distance (*d*_*L*_) as well as the SRMR lead to identical results. The test using the squared Euclidean distance and the SRMR rejects the model somewhat too rarely in case of α = 10% and α = 5% respectively; however, for an increasing sample size, the rejection rates converge to the predefined significance level without reaching it. For the 1% significance level, a similar picture is observed; however, for larger sample sizes, the significance level is retained more often compared to the larger significance levels. In contrast, the test using the geodesic distance mostly rejects the model too often for the 5% and 10% significance level. However, the obtained rejection rates are less often significantly different from the predefined significance level compared to the same situation where the SRMR or the Euclidean distance is used. In case of α = 1% and sample sizes larger than *n* = 100, the test using the geodesic distance rejects the model significantly too often.

**Figure 7:**
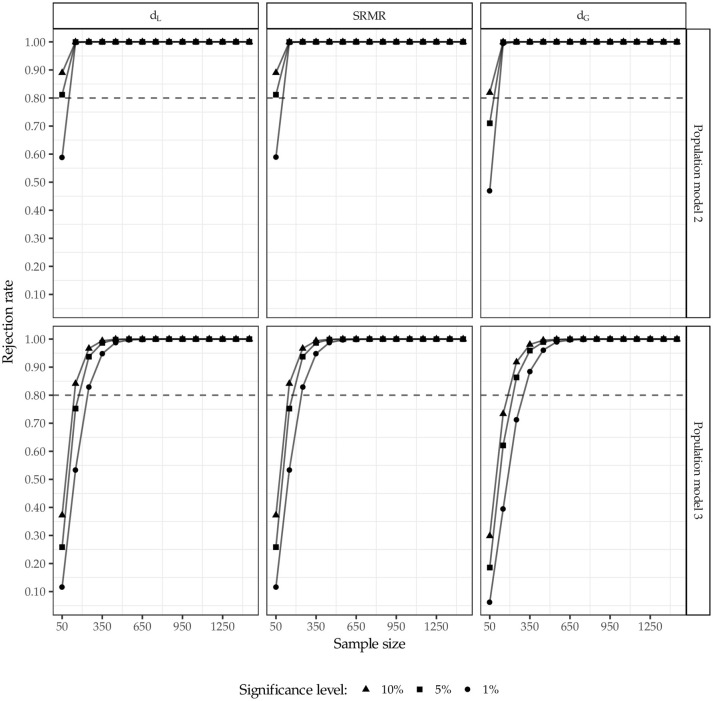
Rejection rates for population model 2 and 3.

Figure 7 displays the rejection rates for population models 2 and 3. The horizontal line at 80% depicts the commonly recommended power for a statistical test (Cohen, 1988). For the two cases where the specification does not match the underlying data generating process, the test using the squared Euclidean distance as well as the SRMR has more power than the test using the geodesic distance, i.e., the test using former discrepancy measures rejects the wrong model more often. For model 2 (confounded indicators) the test produces higher or equal rejection rates compared to model 3 (unexplained correlation). Furthermore, as expected, the power decreases for an increasing level of significance and increases with increasing sample sizes.

**Figure 8:**
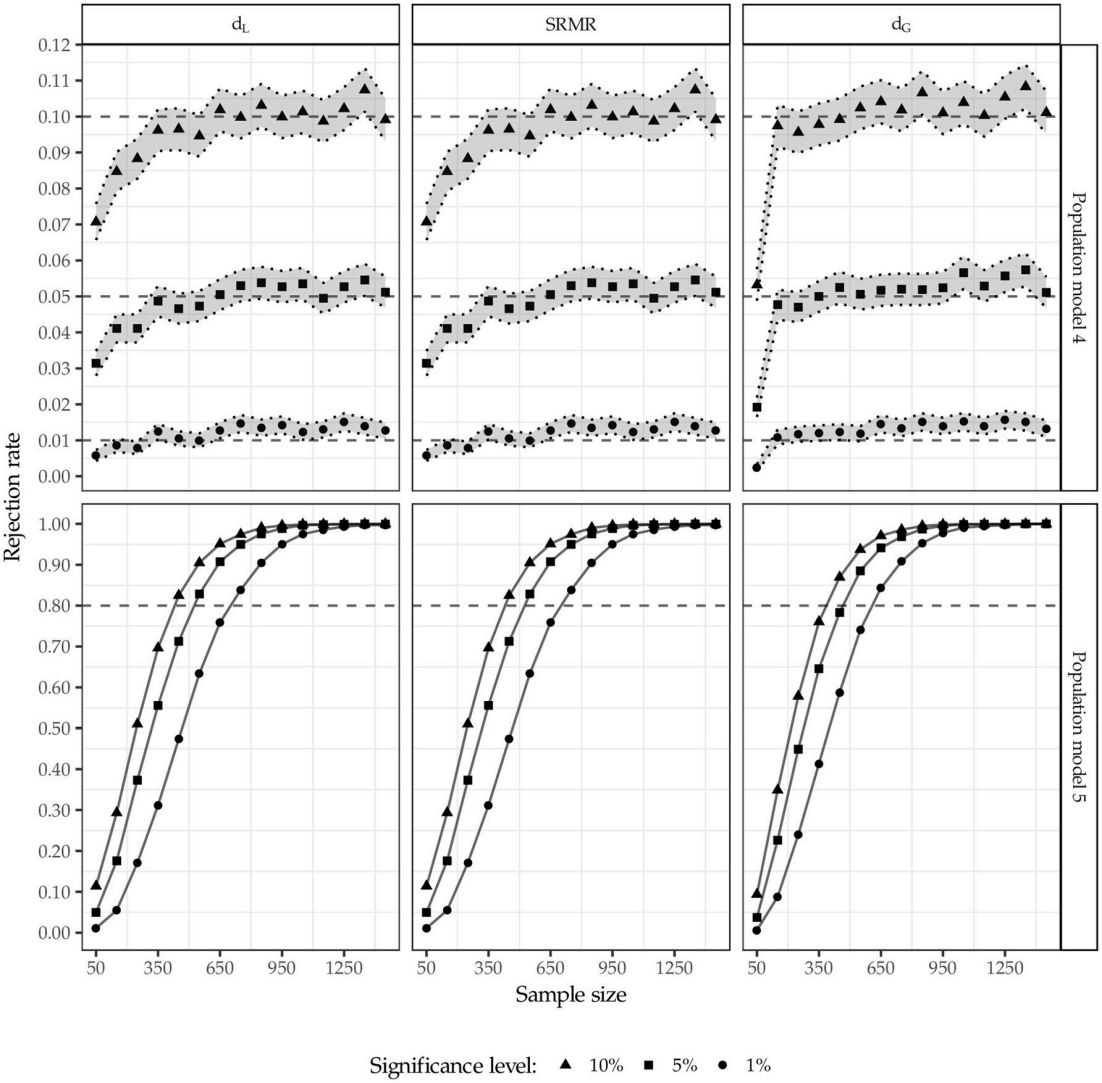
Rejection rates for population model 4 and 5.

Figure 8 depicts the rejection rates for population model 4 and 5. Again, the 95% confidence intervals are illustrated for population model 4 (shaded area) matching the specification estimated. Considering population model 4 which matches the estimated model, the test leads to similar results for all three discrepancy measures. However, the rejection rate of the test using the geodesic distance converges faster to the predefined significance level, i.e., for smaller sample sizes *n* ≥ 100. Again, among the three discrepancy measures considered, the geodesic distance performs best in terms of keeping the significance level.

As the extent of misspecification in population model 5 is minor, the test struggles to detect the model misspecification up to sample sizes *n* = 350, regardless of the discrepancy measure used. However, for sample sizes larger than 350 observations, the test detects the model misspecification satisfactorily. For sample sizes larger than 1,050 observations, the misspecification was identified in almost all cases regardless of the significance level and the discrepancy measure used. Again, this confirms the anticipated relationship between sample size and statistical power.

## 7. Discussion

We introduced the confirmatory composite analysis (CCA) as a full-fledged technique for confirmatory purposes that employs composites to model artifacts, i.e., design concepts. It overcomes current limitations in CFA and SEM and carries the spirit of CFA and SEM to research domains studying artifacts. Its application is appropriate in situations where the research goal is to examine whether an artifact is useful rather than to establish whether a certain concept exists. It follows the same steps usually applied in SEM and enables researchers to analyze a variety of situations, in particular, beyond the realm of social and behavioral sciences. Hence, CCA allows for dealing with research questions that could not be appropriately dealt with yet in the framework of CFA or more generally in SEM.

The results of the Monte Carlo simulation confirmed that CCA can be used for confirmatory purposes. They revealed that the bootstrap-based test, in combination with different discrepancy measures, can be used to statistically assess the overall model fit of the composite model. For specifications matching the population model, the rejection rates were in the acceptable range, i.e., close to the predefined significance level. Moreover, the results of the power analysis showed that the boostrap-based test can reliably detect misspecified models. However, caution is needed in case of small sample sizes where the rejection rates were low, which means that misspecified models were not reliably detected.

In future research, the usefulness of the composite model in empirical studies needs to be examined, accompanied and enhanced by simulation studies. In particular, the extensions outlined by Dijkstra (2017); to wit, interdependent systems of equations for the composites estimated by classical econometric methods (like 2SLS and three-stage least squares) warrant further analysis and scrutiny. Robustness with respect to non-normality and misspecification also appear to be relevant research topics. Additionally, devising ways to efficiently predict indicators and composites might be of particular interest (see for example the work by Shmueli et al., 2016).

Moreover, to contribute to the confirmatory character of CCA, we recommend further study of the performance and limitations of the proposed test procedure: consider more misspecifications and the ability of the test to reliably detect them, find further discrepancy measures and examine their performance, and investigate the behavior of the test under the violation of the normality assumption, similar as Nevitt and Hancock (2001) did for CFA. Finally, cut-off values for the fit indices need to be determined for CCA.

## Author Contributions

FS conducted the literature review and wrote the majority of the paper (contribution: ca. 50%). JH initiated this paper and designed the simulation study (contribution: ca. 25%). TD proposed the composite model and developed the model fit test (contribution: ca. 25%).

### Conflict of Interest Statement

JH acknowledges a financial interest in ADANCO and its distributor, Composite Modeling. The remaining authors declare that the research was conducted in the absence of any commercial or financial relationships that could be construed as a potential conflict of interest.

## Footnotes

^1^For more details and a comprehensive overview, we referred to the following text books: Hayduk (1988), Bollen (1989), Marcoulides and Schumacker (2001), Raykov and Marcoulides (2006), Kline (2015), and Brown (2015).

^2^In general, models containing common factors and composites are also conceivable but have not been considered here.

^3^The existing literature sometimes mentions *empirical (under-)identification* in the context of model identification (Kenny, 1979). Since this expression refers to an issue of estimation rather than to the issue of model identification, this topic is not discussed in the following.

^4^Another way of normalization is to fix one weight of each block to a certain value. Furthermore, we ignore trivial regularity assumptions such as weight vectors consisting of zeros only; and similarly, we ignore cases where intra-block covariance matrices are singular.

^5^The number of degrees of freedom can be helpful at determining whether a model is identified since an identified model has a non-negative number of degrees of freedom.

^6^GCCA builds composites in a way that they are maximally correlated.

^7^For an overview we refer to Kettenring (1971).

^8^In general, GCCA offers several composites (canonical variates); but in our study, we have focused only on the canonical variates of the first stage.

^9^This procedure is known as the Bollen-Stine bootstrap (Bollen and Stine, 1992) in the factor-based SEM literature. The model must be over-identified for this test.

^10^The model-implied correlation between the two indicators is calculated as follows, 0.8 · 0.3 · 0.8 ≠ 0.5.

^11^These functions are provided by the contact author upon request.

^12^The limits of the 95% confidence interval are calculated as, $\hat{p}\pm \Phi^{-1}\left(0.975\right)\sqrt{\hat{p}\left(1-\hat{p}\right)/10000}$, where $\hat{p}$ represents the rejection rate and Φ^−1^() is the quantile function of the standard normal distribution.
