# Supplementary material for: Confirmatory Composite Analysis
Source: Front Psychol. 2018 Dec 13;9:2541. doi: 10.3389/fpsyg.2018.02541 (PMC6300521; doi:10.3389/fpsyg.2018.02541)
Supplement: Supplementary file 1 [file Data_Sheet_1.pdf]

Supplementary Material: Confirmatory Composite Analysis

In the following, we provide a brief example on how to estimate and test a composite model in the statistical programming environment R (R Core Team, 2018) using the cSEM package (Rademaker and Schuberth, 2018). In doing so, we use a simulated dataset based on the population model displayed in Figure S1.

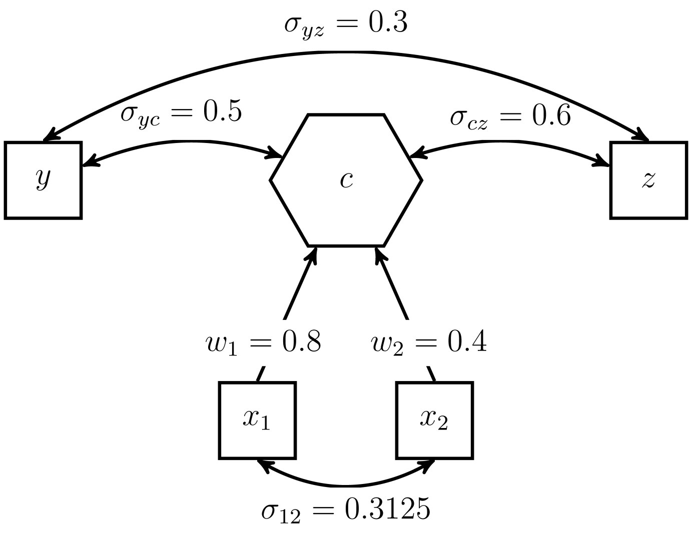

Figure S1. Population model

Before the dataset can be generated, we first read in the indicator correlation matrix of the population model.

```
values = scan(n=16)
1 0.3125 0.4625 0.555
0.3125 1 0.325 0.39
0.4625 0.325 1 0.3
0.555 0.39 0.3 1

nam=c('x1', 'x2', 'y', 'z')
Sigma=matrix(values, ncol = 4, dimnames = list(nam,nam))
```

Second, we draw a dataset from the multivariate normal distribution using the *mvrnorm* function of the MASS package (Venables and Ripley, 2002). In doing so, we set the empirical argument to `TRUE` in order to obtain a dataset with a correlation matrix that equals the population correlation matrix of the indicators.

```
data=MASS::mvrnorm(n = 300, mu = rep(0,4),
                   Sigma = Sigma, empirical = TRUE)
```

Since the cSEM package is currently not available on CRAN, it needs to be installed directly from Github.

```
# Install the cSEM package (currently under development)
devtools::install_github("M-E-Rademaker/cSEM")
library(cSEM)
```

In the next step, the model is defined in *lavaan* syntax (Rosseel, 2012). In doing so, the observable variables y and z are specified as single-indicator composites.

```
# Specification of the model
model='
C <~ x1 + x2
Y <~ y
Z <~ z
Y~C
Z~C
'
```

Finally, the model is estimated and its fit is assessed. Unsurprisingly, the estimated model parameters equal their population counterparts, as the function argument `empirical` of the *mvrnorm* function is set to `TRUE`. Consequently, the bootstrap-based test for overall model does not reject the model as it perfectly fits the dataset.

```
# Estimate the model by the csem function using MAXVAR
out = csem(.data = data, .model = model, .approach_weights = 'MAXVAR')

# Weight estimates
> out$Estimates$Weight_estimates
   x1  x2 y z
C 0.8 0.4 0 0
Y 0.0 0.0 1 0
Z 0.0 0.0 0 1


# model-implied correlation matrix of the indicators
> fit(out)
       x1     x2      y     z
x1 1.0000 0.3125 0.4625 0.555
x2 0.3125 1.0000 0.3250 0.390
y  0.4625 0.3250 1.0000 0.300
z  0.5550 0.3900 0.3000 1.000
```

```
# test the overall model fit
testout = testOMF(out, .saturated = TRUE)

> testout
________________________________________________________________________________
-------- Test for overall model fit based on Dijkstra & Henseler (2015) -----

Null hypothesis:

           +----------------------------------------------------------+
           |                                                          |
           |   H0: No significant difference between empirical and   |
           |   model-implied indicator covariance matrix.            |
           |                                                          |
           +----------------------------------------------------------+

Test statistic and critical value:

                                   Critical value
Distance measure    Test statistic   95%
dG                      0.0000     0.0022
SRMR                    0.0000     0.0242
dL                      0.0000     0.0029


Decision:

                     Significance level
Distance measure          95%
dG                   Do not reject
SRMR                 Do not reject
dL                   Do not reject

Additonal information:

Out of 499 bootstrap replications 499 are admissible.
See ?verify() for what constitutes an inadmissible result.
________________________________________________________________________________
```

REFERENCES

R Core Team (2018). *R: A Language and Environment for Statistical Computing*. R Foundation for Statistical Computing, Vienna, Austria

Rademaker, M. and Schuberth, F. (2018). *cSEM: Composite-Based Structural Equation Modeling*. R package version 0.0.0.9000

Rosseel, Y. (2012). lavaan: An R package for structural equation modeling. *Journal of Statistical Software* 48, 1–36

Venables, W. N. and Ripley, B. D. (2002). *Modern Applied Statistics with S* (New York: Springer), 4th edn.
